# Understanding vaccination gaps in Peru: a convergent cross-sectional analysis of coverage, parental perceptions, and child mortality data in Sunampe

**DOI:** 10.3389/fpubh.2026.1779442

**Published:** 2026-08-26

**Authors:** Margarita Norma Castro-Fuentes, Carlos Andrés Mugruza-Vassallo

**Affiliations:** 1 School of Nursing, Faculty of Health Sciences, Universidad Privada San Juan Bautista (UPSJB), Chincha, Peru; 2 Grupo de Investigación en Computación y Neurociencia Cognitiva, Universidad Tecnológica de Lima Sur (UNTELS), Lima, Peru

**Keywords:** anemia, respiratory mechanics, COVID-19, health literacy, parental knowledge, machine learning, vaccine compliance, vaccine-preventable diseases

## Abstract

**Background:**

The Sunampe district on the Peruvian coast faces challenges related to adherence to childhood vaccination schedules. This study aimed to assess whether non-compliance with vaccination correlates with an increased risk of vaccine-preventable epidemiological vulnerability and health system fragility among children under five, within the context of low coverage in Sunampe.

**Methods:**

An analytical, correlational, cross-sectional study was conducted involving 123 families in Sunampe. Data on vaccination status, reported illnesses, and mortality of children aged 1 to 5 years were collected. Medical records were reviewed for vaccine-preventable diseases, and mortality data were obtained from the SINADEF database. Severe non-compliance was defined as vaccination discontinued by 6 months of age (≥3 missed key doses). Logistic regression with prespecified predictors (knowledge, fear, access) was used to identify determinants of severe non-compliance. Mortality data were obtained from the SINADEF database (2017–2022). Sensitivity analysis using Firth’s penalized logistic regression with dimension reduction (composite knowledge, fear, and access scores) addressed low events-per-variable (EPV = 4.5).

**Results:**

Some Peruvian districts had to improve health facilities, while families in Sunampe perceived the single local health center as insufficient. No active vaccine-preventable illnesses were reported among surveyed children, but SINADEF data showed 10 deaths in children under five between 2017 and 2022, including 6 around respiratory mechanics issues (Pneumococcus) and 1 with malnutrition (no clearly anemia). Also, it was shown the prevalence of illness and incomplete immunization, as well as increased rates of anemia in Peru. Logistic regression models had fair to good predictive power and identified two significant predictors of severe non-compliance: lack of knowledge about vaccination age (Q17: OR = 10.96, 95% CI: 3.40–35.36, *p* = 0.0001) and never attending a vaccine information session (Q18: OR = 3.32, 95% CI: 1.16–9.57, *p* = 0.026). Fear of reactions (reported by 99.2% of mothers) did not reach statistical significance. The model showed good discriminative ability (AUC = 0.852, accuracy = 76.4%). Firth penalized logistic regression on composite scores confirmed knowledge as the only significant predictor (OR = 3.84 per 1-point increase, *p* < 0.001), while fear (OR = 2.82, *p* = 0.082) and access (OR = 0.80, *p* = 0.512) were not significant.

**Conclusion:**

Incomplete vaccination in Sunampe appears linked to parental knowledge gaps and concerns about vaccine side effects, despite most parents reporting no severe reactions. Fear of reactions did not predict behavior, suggesting that reported safety concerns may reflect social desirability bias rather than true barriers. These findings highlight the need, in clinical decision-making, for targeted education to improve vaccination adherence and reduce epidemiological vulnerability and health system fragility.

## Introduction

Immunization remains a cornerstone of public health, particularly for children under 5 years of age. Despite its proven efficacy, millions of children globally remain at risk for diseases such as diphtheria, pertussis, and tetanus due to incomplete or delayed vaccination coverage. According to the World Health Organization (1), these vaccine-preventable diseases (VPDs) continue to pose serious threats to child health. Hernández-Ávila (2) reported that in 2021, 25 million children missed one or more doses of the DPT (diphtheria, pertussis, and tetanus) vaccine—an alarming rise from 19 million in 2019.

The COVID-19 pandemic further disrupted routine immunization programs, intensifying challenges such as vaccine hesitancy and misinformation (3–5). A historical example illustrating the consequences of declining vaccine coverage occurred in Russia, where diphtheria cases surged from 1,200 in 1990 to 5,000 in 1993 following reduced immunization rates (6). Similarly, pertussis has shown changing epidemiological patterns in recent years, with fluctuations associated with pandemic-era healthcare disruptions (3) and parental concerns about vaccine safety (4, 5).

Misinformation has also played a critical role in undermining vaccine efforts. For instance, in Nigeria, false claims about the polio vaccine causing infertility led to the suspension of immunizations in 2003 and triggered a polio outbreak the following year in which 70% of affected children suffered paralysis (7). In Peru, similar concerns have emerged, especially after nearly four decades without polio cases. The Working Subgroup for Immunizations (7) has recently issued warnings about the possibility of resurgence due to gaps in vaccination coverage.

Invasive bacterial infections (IBIs) remain another concern, particularly among children with sickle cell disease (SCD). Major pathogens such as *Streptococcus pneumoniae*, *Haemophilus influenzae* type b (Hib), and non-typhoidal Salmonella species are leading causes of morbidity and mortality among children under five, especially in low-resource settings (8).

Despite regional progress, outbreaks have re-emerged in countries once considered measles-free. In Argentina, for example, a measles outbreak between 2018 and 2019 was attributed to insufficient vaccination coverage (9). Before the introduction of the measles vaccine in 1963, the disease caused an estimated 2.6 million deaths annually, with epidemics every 2 to 3 years (9). In Peru, Luna et al. (10) found that the introduction of the PCV7 vaccine significantly reduced invasive pneumococcal disease in children under two, though ongoing monitoring is necessary to track serotype shifts and antibiotic resistance. Globally, pneumococcal infections remain a leading cause of death among young children, claiming nearly half a million lives annually—mostly in low- and middle-income countries (10).

Operational challenges also hinder effective immunization. In Lao PDR, studies by Nanthavong et al. (11) and Xaydalasouk et al. (12) identified issues in vaccine distribution, cold chain maintenance, and limited outreach in ethnically diverse or underserved regions. Vaccines prevent more than 20 serious diseases, including tetanus, influenza, pneumonia, measles, and severe diarrhea, all of which are particularly dangerous in early childhood (12).

Integrated strategies have proven effective in improving immunization outcomes. In Ethiopia, Abrha et al. (13) demonstrated that combining vaccinations with vitamin A supplementation improved both immune function and infection resistance in children, with 92.4% of participants receiving full vaccinations and 85.2% receiving supplementation.

In Peru, the National Centre for Epidemiology, Prevention, and Disease Control (CDC-MINSA) (14) recorded 1,661 measles cases and 45 related deaths in children under five between 2016 and 2021. A diphtheria outbreak was also reported in 2020, breaking a two-decade absence (15). These developments underscore the persistent vulnerability of children to VPDs, especially in areas with low immunization rates such as Sunampe (16).

Documentation and record-keeping further complicate disease prevention. In Italy, some children were considered vaccinated after receiving two doses at least 4 weeks apart, even in the absence of prior records (17). Similarly, a study by Xu et al. (18) on child mortality in China overlooked vaccination status as a potential factor, highlighting the need for more comprehensive assessments of immunization in health outcomes research.

Latin American countries, including Argentina, Chile, Mexico, Brazil, Venezuela, Colombia, and Ecuador, continue to struggle with consistent vaccine coverage, enabling the circulation of preventable pathogens and increasing regional public health risks (1). In this context, children in areas like Sunampe, Peru, remain especially vulnerable due to poor access, inadequate infrastructure, and rising parental skepticism.

Immunization coverage rates often mask substantial delays; many children receive vaccines weeks or months after the recommended age, leaving a prolonged window of susceptibility (19, 20). Delayed administration of BCG, DPT, and measles has been associated with lower maternal education, poorer household wealth, and home birth in large-scale Choudhary’s surveys from India (21). Moreover, EPI has averted over 150 million deaths globally since 1974, with 40% of the decline in infant mortality attributable to vaccination (22), underscoring that both timeliness and equity remain critical.

In Sunampe, the risk of VPD outbreaks coincides with low vaccination coverage and a growing number of susceptible children. According to the Chincha Health Executive Unit (23), the district is served by only one health facility, placing significant strain on resources and limiting access to timely immunizations.

This study seeks to evaluate the risk of VPD outbreaks in children under five in Sunampe, Peru, by analyzing vaccination compliance, reported illnesses, and mortality data. Specifically, it addresses the question: Is there a possibility of an outbreak of vaccine-preventable diseases among children under five linked to low vaccination coverage and non-compliance in Sunampe, Peru?

## Materials and methods

### Study design

**Figure 1:**
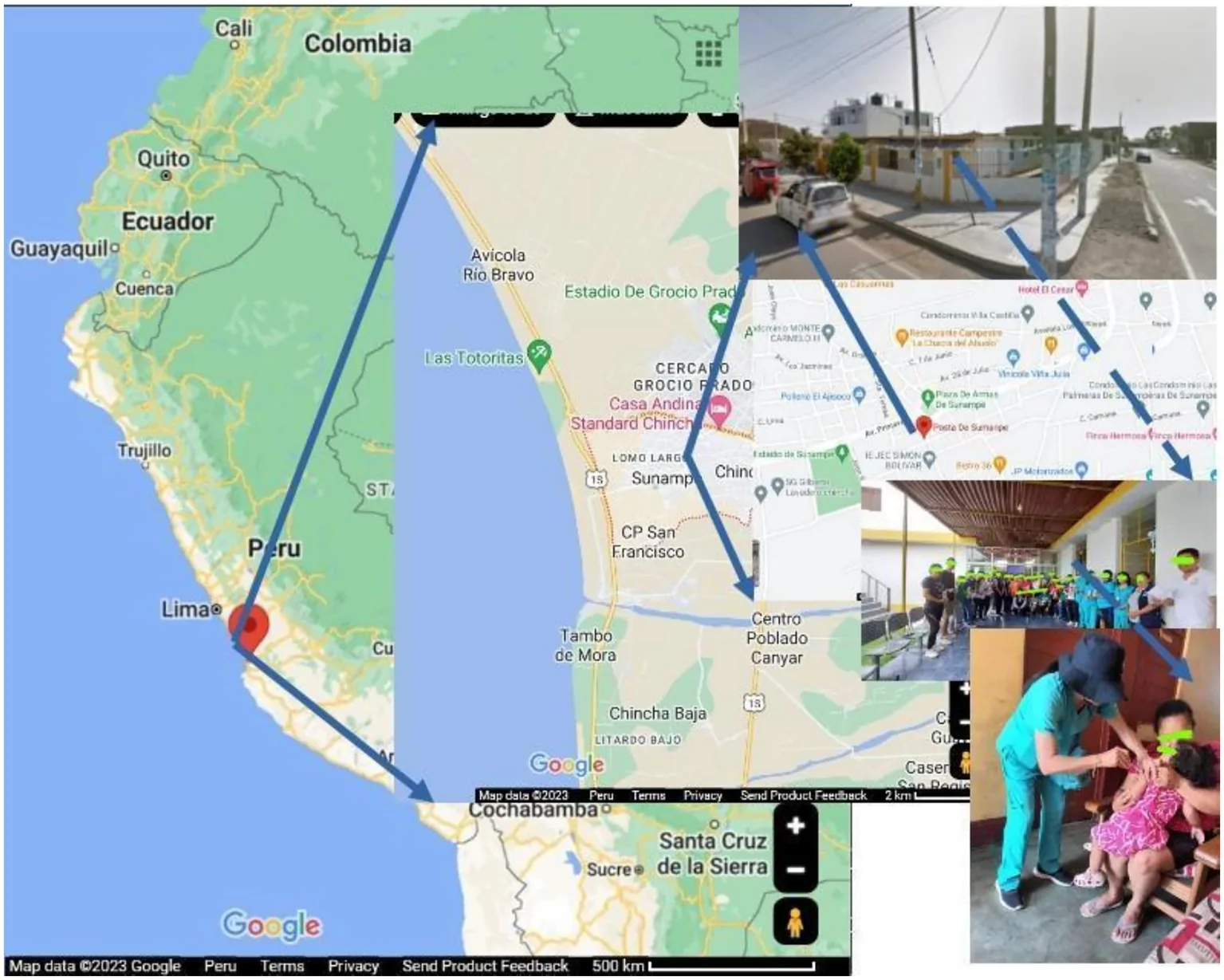
Sunampe medical facility (*Posta*), vaccine team, vaccination and geographical position: −13.427796195563445, −76.16436904957237. Figure processed from GoogleMaps for Health Centre at Sunampe on March 2023. Photo from October 2023.

**Figure 2:**
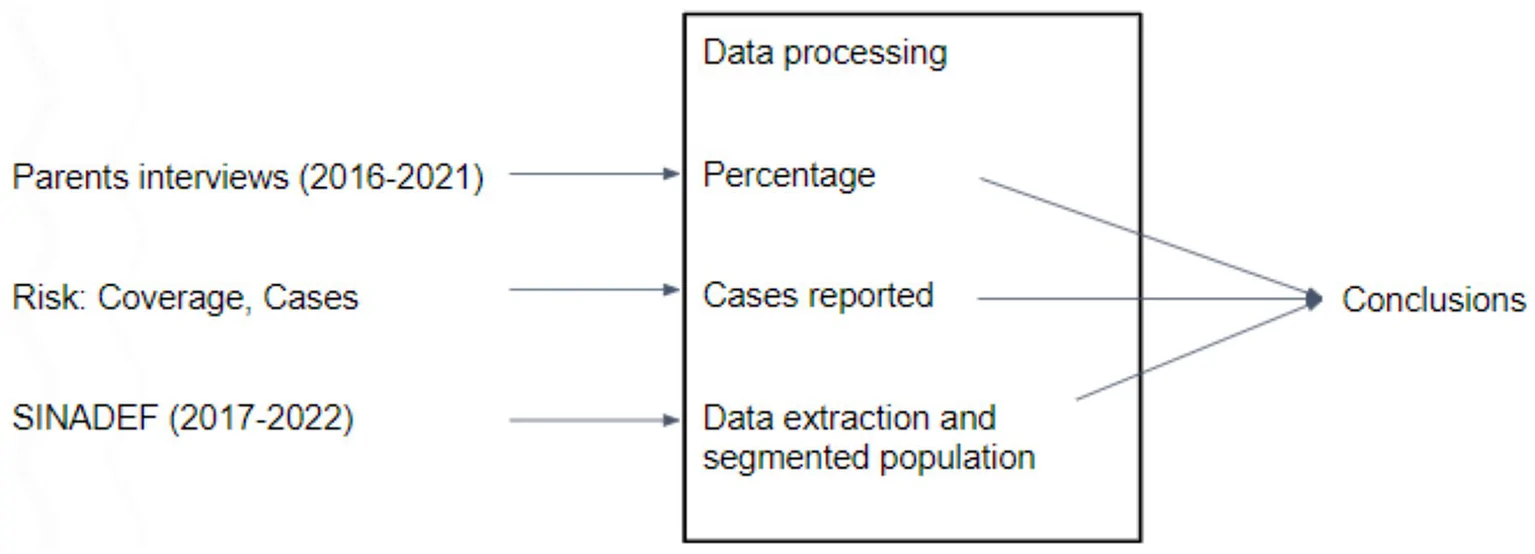
Block diagram of data processing in the overall study.

This study employed quantitative, observational, and analytical methods to assess epidemiological vulnerability associated with non-compliance with vaccination schedules and low coverage in the Sunampe district of Chincha province, Peru (see Figure 1). The study comprised three components: Vaccination, Patient Cases, and Deaths in Sunampe. Ethical approval was obtained under the research projects “Riesgo de brote de enfermedad inmunoprevenible en niños menores de cinco años relacionado con incumplimiento de vacunación y bajas coberturas Sunampe” (2019-2019-UPSJB) for parent interviews and “Caracterización de causas de los niños de hasta 5 años fallecidos en SUNAMPE” (0809-2023-CIEI-UPSJB) for final data compilation (see Figure 2 for data processing details).

### Study population and data collection

From a registry of 560 children with incomplete vaccine records, we prioritized those 180 with missing key vaccines. Of these, 123 families completed the full interview and were included in the final analysis. Then, the study included 123 children under 5 years of age (66 males, aged 4–59 months), selected through simple random probabilistic sampling of families using the ESSALUD or SIS Peruvian public health systems. Permission for data collection was obtained from the Sunampe district health center. Medical records were reviewed to assess vaccination coverage over a 5-year period (2017 to 2021), focusing on the nominal registry of children under five in Sunampe. Clinical trial number: not applicable.

From incomplete vaccinations, priority was given to those missing key vaccines: APO (oral antipolio), Pentavalent (DPT-HepB-Hib), Pneumococcus, SPR (measles, mumps, and rubella), and DPT (diphtheria, pertussis, and tetanus) doses 1 and 2. Inclusion criteria required children to reside in Sunampe, have initiated their vaccination schedule at the district health center, and be between 4 and 59 months old. Non-priority vaccines, such as AMA, chickenpox, and seasonal influenza, were excluded.

To explore reasons for non-compliance with vaccination schedules, a 25-question survey (Table 1) was administered to mothers of children under five. The questionnaire, validated through expert judgment and pilot testing, achieved a reliability score of 0.75 (KR20). It assessed maternal awareness, accessibility, and perceptions of vaccine reactions to identify factors contributing to non-compliance. Also contextual observations were given by mothers during the survey.

**Table 1:** Survey questions.

Question code	Survey question
Q01	Has your child received any vaccinations?
Q02	Did your child get vaccinated only up to 2 months?
Q03	Did your child get vaccinated only up to 4 months?
Q04	Did your child get vaccinated only up to 6 months?
Q05	Did your child get vaccinated only up to 1 year?
Q06	Has your child had any disease that is prevented by vaccines?
Q07	Do you live near the health center?
Q08	Are the vaccines free of charge at the health center?
Q09	Did you find it easy to get vaccinated at the Health center?
Q10	Have you always found vaccines at the health center?
Q11	Do you spend a long time waiting to get vaccinated?
Q12	Are the waiting times for vaccination not convenient for you?
Q13	Is the number of vaccination centers in your district sufficient?
Q14	Do you know how many immunizations through vaccines your child should have?
Q15	Do you understand the importance of vaccines?
Q16	Do you know what diseases each of your child’s vaccines protects against?
Q17	Do you know at what age your child should be vaccinated?
Q18	Have you ever attended a vaccine information session?
Q19	Are you aware of the risk of developing a disease that can be preventable by vaccines?
Q20	Has your child had a serious reaction to a vaccine?
Q21	Are you worried about reactions to vaccines?
Q22	Does anyone in your family object to your child being vaccinated?
Q23	Is the vaccine reaction the reason for not vaccinating your child?
Q24	Do you belong to a religion that forbids vaccination?
Q25	Has your child ever had an allergic reaction to a vaccine?

The World Health Organization’s Expanded Programme on Immunization (EPI) recommends that the primary series of pentavalent and polio vaccines be completed by 6 months of age. Missing ≥3 key doses by this age constitutes failure to complete the critical primary series, leaving infants unprotected during the highest-risk period for pertussis, diphtheria, and *Haemophilus influenzae* type b (Hib) disease (21, 24).

Delayed vaccination increases the susceptibility window for vaccine-preventable diseases, and high coverage rates do not guarantee age-appropriate vaccination (19, 20, 25, 26). In particular, delayed administration of Bacille Calmette-Guérin (BCG) during the first 6 months of life has been associated with reduced survival (24), and delayed DPT/measles doses are consistently linked to lower wealth, lower maternal education, and home birth (21).

Therefore, severe non-compliance was defined *a priori* as discontinuation of vaccination by 6 months of age with ≥3 missed key doses (OPV, pentavalent, PCV, MMR, or DPT). This definition aligns with international EPI milestones and recognized risk factors for delayed immunization. The alternative 4-month threshold (≥2 missed doses) provided poorer group balance (54 severe vs. 69 mild) and identified only one significant predictor (knowledge of vaccination age), whereas the 6-month threshold (45 severe vs. 78 mild) identified two actionable predictors (knowledge of vaccination age and attendance at information sessions).

### Data sources and processing

Data from the National Information System of Deaths (SINADEF) were processed to extrapolate findings from the Institute for Health Metrics and Evaluation (IHME) Global Burden of Disease (GBD) 2017 (27) dataset on children under five. This was merged with SINADEF data from 2017 to 2022, accessed via the Peruvian Ministry of Health’s open data portal^1^. Pre-processed data are available on GitHub^2^ (see Figure 2).

Given the low mortality rate in Sunampe, national data from all regions of Peru were also analyzed. Data were processed using Octave and MATLAB, with filtered SINADEF data reviewed independently by two reviewers to identify vaccine-preventable diseases in children under five. To access this segment of data, one may refer to the virtual repository on GitHub titled ‘Sunampe.mat’.

### Risk assessment: coverage, cases, deaths

Susceptibility to vaccine-preventable diseases was calculated as the sum of unvaccinated and non-immunized individuals:

$$\begin{array}{l} \text{Total susceptibility}=\text{number of unvaccinated}+\\ \text{number of non}-\text{immunized} \end{array}$$

Public data from the National Centre for Epidemiology, Prevention, and Control of Diseases of Peru were analyzed to compare vaccination trends in Ica and the country as a whole. Differences in SPR1 and SPR2 vaccination coverage between 2015 and 2020 were also examined (see Figure 2).

### Statistical analysis

The primary analysis focused on identifying factors associated with binary vaccination outcomes. Logistic regression analysis was used as a baseline supervised machine learning technique. Here we employed to model relationships between parental concerns and access barriers (independent variables) and vaccination outcomes (dependent variables). See details in Supplementary material.

Before running the models, the dataset was pre-processed to handle missing values: numerical variables were imputed with the mean and categorical variables with the mode. Predictor variables exhibiting perfect or near-perfect prediction of the outcome were excluded to avoid quasi-separation issues.

A single pre-specified logistic regression model was constructed with ‘incomplete vaccination status (missing ≥1 age-appropriate dose)’ as the primary outcome. Predictors were selected *a priori*: (1) maternal knowledge, (2) fear of reactions, (3) access (wait time, distance). No stepwise selection was used (threshold: *p* < 0.05).

Model 1 (Annex–4-month threshold):

log(p/(1-p)) = −22.69 + 1.09(Q14) + 11.48(Q16) + 1.94(Q17) + 1.07(Q18) + 2.42(Q20) - 1.77(Q21) + 19.50(Q23) + 0.72(Q25) - 0.37(Q07) + 1.27(Q09) + 0.73(Q11) + 2.92(Q12) + 0.91(Q13).

Model 2 (Main – 6-month threshold):

log(p/(1-p)) = −22.18 + 0.57(Q14) + 2.40(Q17) + 1.20(Q18) + 1.05(Q20) – 0.04(Q25) – 0.62(Q07) + 0.86(Q09) – 0.19(Q11) + 1.85(Q12) – 0.54(Q13).

Handling of low events-per-variable (EPV). The primary model contained 45 severe non-compliance events and 10 candidate predictors (EPV = 4.5), below the recommended minimum of 10 by Peduzzi (19). To address this limitation and stabilize odds ratio estimates, we conducted a sensitivity analysis using Firth’s penalized logistic regression (20) with dimension reduction, as the supervised machine learning technique for robust binary classification. Individual questionnaire items were aggregated into three composite scores based on clinical domain:

6-month threshold (main):

log(p/(1-p)) = −3.68 + 1.35(Knowledge) + 1.04(Fear) – 0.23(Access)*.

4-month threshold (Annex):

log(p/(1-p)) = −3.01 + 1.36(Knowledge) + 1.28(Fear) - 0.34(Access).

This method constructed explanatory models by selecting significant predictors from the remaining items. Model performance was evaluated using the Akaike Information Criterion (AIC), Area Under the Receiver Operating Characteristic Curve (AUC), and classification accuracy. Additional tools included confusion matrices, classification reports, and ROC curves.

Based on the primary inference is based on the forward prespecified model (Supplementary Table S1), not on the exploratory analysis, an exploratory inter-item association analyses (Supplementary Table S2) were conducted to identify potential hub variables and inform the selection of prespecified predictors for the main logistic regression model.

All statistical analyses were conducted using Python libraries (pandas, numpy, statsmodels). SINADEF data were processed with Excel, Octave, and MATLAB. Only factors independently associated with incomplete or absent diphtheria vaccination in Sunampe PDR were included in the final analysis.

## Results

### Population characteristics

As shown in Table 2A, 93.5% of mothers interviewed had public insurance coverage (SIS or ESSALUD), and 89.4% were in a relationship (either married or cohabiting). Of the children surveyed, 53.7% were male.

**Table 2:** Population compliance, access, mother’s knowledge to the risk–benefit, with the vaccination schedule and overall summary in the 123 interviews.

**(A) Sociodemographic table**			
		Number	Percentage
Children gender	Male	66	53.7%
	Female	57	46.3%
Safe Child Type	Public	115	93.5%
	Private	7	5.7%
	Do not have	1	0.8%
Mother Civil Status	Single	13	10.6%
	Married	49	39.8%
	Cohabiting	61	49.6%
	Coexistent	0	0.0%

(B) Vaccination status							
		Has your child received any vaccinations?	Did your child get vaccinated only up to 2 months?	Did your child get vaccinated only up to 4 months?	Did your child get vaccinated only up to 6 months?	Did your child get vaccinated only up to 1 year?	Has your child had any disease that is prevented by vaccines?
*n* = 123	Yes	122	62	45	57	57	123 (no one)
	No	1	61	78	66	66	0
Mean		0.99	0.50	0.63	0.46	0.46	0.01
Median		1.00	1.00	1.00	0.00	0.00	0.00
Variance		0.008	0.252	0.234	0.251	0.251	0.008

**(C)** Access to the vaccine								
		Do you live near the health centre?	Are the vaccines free of charge at the health centre?	Did you find it easy to get vaccinated at the health centre?	Have you always found vaccines at the health centre?	Do you spend a long time waiting to get vaccinated?	Are the waiting times for vaccination not convenient for you?	Is the number of vaccination centers in your district sufficient?
*n* = 123	Yes	50	113	21	77	112	110	6
	No	73	10	102	46	11	13	117
Mean		0.41	0.92	0.17	0.63	0.09	0.89	0.05
Median		0.00	1.00	0.00	1.00	0.00	1.00	0.00
Variance		0.243	0.075	0.143	0.236	0.082	0.095	0.047

(D) Mother’s knowledge							
		Do you know how many immunizations through vaccines your child should have?	Do you understand the importance of vaccines?	Do you know what diseases each of your child’s vaccines protects against?	Do you know at what age your child should be vaccinated?	Have you ever attended a vaccine information session?	Are you aware of the risk of developing a disease that can be preventable by vaccines?
*n* = 123	Yes	15	120	1	69	36	45
	No	108	3	122	54	87	78
Mean		0.12	0.98	0.01	0.56	0.29	0.37
Median		0.00	1.00	0.00	1.00	0.00	0.00
Variance		0.108	0.024	0.008	0.248	0.209	0.234

**(E)** Vaccine reaction							
		Has your child had a serious reaction to a vaccine?	Are you worried about reactions to vaccines?	Does anyone in your family object to your child being vaccinated?	Is the vaccine reaction the reason for not vaccinating your child?	Do you belong to a religion that forbids vaccination?	Has your child ever had an allergic reaction to a vaccine?
*n* = 123	Yes	4	122	68	120	7	20
	No	119	1	55	3	116	103
Mean		0.03	0.99	0.55	0.98	0.06	0.16
Median		0.00	1.00	1.00	1.00	0.00	0.00
Variance		0.032	0.008	0.249	0.024	0.054	0.137

(F) Vaccination coverage 2017–2021 Chincha Province and Sunampe District Extracted and adapted from the Office of Telecommunications and Information Technology of the Executing Unit 401 S-Chincha.						
	Chincha Province–type of vaccine			Sunampe District–type of vaccine		
Group	> 1 year	1 year	4 year	> 1 year	1 year	4 year
Vaccine	PENTA 3	SPR 2	APO 2	APO 3	DPT 1	APO 2
2017	86.88%	62.77%	23.94%	93.29%	65.63%	22.84%
Vaccine	APO 3	DPT 1	APO 2	APO 3	DPT 1	APO 2
2018	83.04%	68.12	69.39%	63.87%	64.26%	56.76%
Vaccine	PENTA 3	SPR 2	DPT 2	PENTA 3	SPR 2	DPT 2
2019	83.43%	72.65%	57.38%	60.90%	63.83%	54.02%
Vaccine	APO 3	SPR 2	APO 2	APO 3	SPR 2	APO 2
2020	72.31%	47.59%	64,25%	55.45%	43.17%	65.17%
Vaccine	APO 3	SPR 2	APO 2	APO 3	SPR 2	APO 2
2021	81.49%	49.38%	45.59%	55.40%	53.85%	43,89%

### Vaccination

All 123 children included in the study exhibited some form of non-compliance with the official vaccination schedule. Despite this, Table 2B indicates that none had developed pneumonia, diphtheria, diarrhea, or any other vaccine-preventable disease. However, adverse reactions to vaccines were reported in several cases, contributing to delayed or irregular vaccination schedules.

In many instances, vaccination was initiated but interrupted, often after the two-month immunization visit. For example:

Case 1: A child received all vaccines at 2 months but did not return to the health facility until age one. At that point, only the vaccines with the least reported side effects were administered in order to encourage the mother to return and complete the immunization schedule.

Case 2: Another child arrived at the health center at age one having only received neonatal vaccines. The mother expressed fear of vaccine reactions. Consequently, only the age-appropriate vaccines not requiring previous doses—such as MMR (SPR), varicella, and influenza—were administered. The mother was advised to return in 2–3 weeks to continue the schedule.

These cases exemplify the pattern of partial immunization and parental hesitancy. Of the children assessed, 24 were under 1 year of age, and 99 were older than 1 year (32 aged one, 17 aged two, 21 aged three, and 29 aged four). Among the 99 children older than 1 year, only 57 had received the full vaccine series appropriate for their age.

Due to the existence of only one public health facility in Sunampe, 59.35% (73/123) of respondents reported that they lived far from the center (Table 2C). Some families opted to visit health posts in neighboring districts such as Chincha Baja, Grocio Prado, and Pueblo Nuevo due to more accessible transportation or shorter wait times. These findings underscore that geographic access remains a substantial barrier to full vaccination compliance.

Regarding service delivery, long wait times were a commonly reported barrier. Due to the computerized appointment system, up to six families per day brought their children without prior appointments, often waiting between 2 and 4 h. Table 2C confirms that 91.06% (112/123) of respondents experienced these long wait times.

Despite this, 89.43% (110/123 in Table 2D) of parents felt that the opening hours of the health facility were adequate, although only 4.88% (6/123) considered the number of health centers sufficient. These results suggest a public perception that additional health facilities are needed to improve access and timely vaccination services for children.

### Knowledge and risk perception

Table 2D highlights major informational gaps. While 97.56% (120/123) of mothers acknowledged the importance of vaccines in preventing disease, 87.80% (108/123) did not know how many vaccines their child should receive, and 99.19% (122/123) were unaware of which diseases vaccines protect against. Furthermore, 70.73% (87/123) had not attended any educational or informational session on childhood immunization. Only 36.59% (45/123) were aware of the risk of contracting vaccine-preventable diseases. This discrepancy suggests that while general support for vaccination exists, detailed knowledge remains limited—potentially contributing to hesitancy and non-compliance.

### Vaccine reactions and parental concerns

As shown in Table 2E, only 3.25% (4/123) of respondents reported that their child experienced a serious vaccine-related reaction. However, 99.19% (122/123) of mothers expressed fear of potential side effects, and 68 mothers (55.28%) indicated that a family member opposed vaccination. In addition, 120 mothers (97.56%) identified fear of adverse reaction as the primary reason for not vaccinating their child, despite the absence of serious reactions in most cases.

Religious or medical contraindications (Table 2E) were rare: only 5.69% (7/123) cited religious prohibition, and 16.26% (20/123) noted that their child had experienced an allergic or mental health-related reaction. These data point to perceived fear—rather than actual adverse events—as the principal driver of vaccine non-compliance. This highlights the urgent need for community-wide information campaigns to reduce misconceptions and encourage full immunization.

Children missing critical vaccine doses, such as APO2 and PENTA3, further confirm low immunization coverage in the district (see Table 2F), reinforcing concerns raised by national data processed by MINSA.

### Results of logistic regression analysis

#### Severity of non-compliance

Among the 123 children included in the study, 45 (36.6%) met criteria for severe non-compliance (vaccination discontinued by 6 months of age, defined as missing ≥3 key vaccine doses), while 78 (63.4%) had mild non-compliance (missing 1–2 doses).

#### Logistic regression analysis

A single pre-specified logistic regression model was constructed to identify predictors of severe non-compliance (vs mild non-compliance). Table 3 (Model 2) presents the final logistic regression model for severe non-compliance defined as vaccination stopped by 6 months (≥3 missed doses). A comparison with the 4-month threshold model is provided. Predictors were selected *a priori* from three conceptual domains: parental knowledge (Q14, Q16, Q17, Q18), fear of reactions (Q20, Q21, Q23, Q25), and access barriers (Q07, Q09, Q11, Q12, Q13). Due to perfect separation causing model non-convergence, three predictors (Q16, Q21, Q23) were excluded from the final model.

**Table 3:** Logistic regression models predicting severe vaccination non-compliance at two thresholds (*n* = 123).

**Predictor**	**Model 1: 4-month threshold (Annex)**		**Model 2: 6-month threshold (Main)**	
	OR [95% CI]	*p*-value	OR [95% CI]	*p*-value
Knowledge domain				
Knows number of vaccines (Q14)	2.99 [0.50–17.90]	0.231	1.77 [0.39–8.00]	0.457
Knows vaccines/diseases (Q16)†	96,616 [0.00 – ∞]	0.983	— (excluded)	—
Knows vaccination age (Q17)	**6.98 [2.38–20.45]**	**0.0004**	**10.96 [3.40–35.36]**	**0.0001**
Attended info session (Q18)	2.92 [0.99–8.64]	0.053	**3.32 [1.16–9.57]**	**0.026**
Fear domain				
Child had serious reaction (Q20)	11.28 [0.76–168.06]	0.079	2.86 [0.19–43.19]	0.449
Worried about reactions (Q21)†	0.17 [0.00 – ∞]	1	— (excluded)	—
Reaction reason for not vaccinating (Q23)†	2.96 × 10^8^ [0.00 – ∞]	0.999	— (excluded)	—
Child had allergic reaction (Q25)	2.05 [0.60–7.01]	0.253	0.96 [0.28–3.28]	0.951
Access domain				
Lives near health center (Q07)	0.69 [0.25–1.90]	0.476	0.54 [0.20–1.46]	0.225
Easy to get vaccinated (Q09)	3.57 [0.91–14.03]	0.068	2.37 [0.70–8.08]	0.167
Long waiting time (Q11)	2.08 [0.32–13.56]	0.444	0.83 [0.15–4.53]	0.83
Waiting time inconvenient (Q12)	18.56 [0.78–439.26]	0.07	6.34 [0.42–96.53]	0.184
Sufficient vaccination centers (Q13)	2.50 [0.22–28.31]	0.46	0.58 [0.08–4.29]	0.597

Model summary	Model 1 (Annex)		Model 2 (Main)	
Severe non-compliance definition	Stopped by 4 months (≥2 missed doses)		Stopped by 6 months (≥3 missed doses)	
Severe non-compliance (*n*, %)	54 (43.9%)		45 (36.6%)	
Mild non-compliance (*n*, %)	69 (56.1%)		78 (63.4%)	
AUC	0.866		0.852	
Accuracy	80.50%		76.40%	
Pseudo *R*^2^	0.33		0.301	
Convergence	Failed (perfect separation)		Failed (perfect separation)	
Excluded predictors	—		Q16, Q21, Q23	

^†^IC is not consistent, therefore excluded for Model 2 in regression analysis. Bold highlights significant values.

The model demonstrated good discriminative ability (AUC = 0.852, accuracy = 76.4%). Two predictors achieved statistical significance (*p* < 0.05):

Q17 (“Do you know at what age your child should be vaccinated?”): OR = 10.96 (95% CI, 3.40–35.36, *p* = 0.0001).

Q18 (“Have you ever attended a vaccine information session?”): OR = 3.32 (95% CI, 1.16–9.57, *p* = 0.026).

Parents who did not know the appropriate vaccination age were nearly 11 times more likely to discontinue vaccination by 6 months. Additionally, parents who had never attended a vaccine information session were 3.3 times more likely to have severely non-compliant children.

Other predictors, including fear of adverse reactions (Q20, Q25) and access barriers (Q07, Q09, Q11, Q12, Q13), did not reach statistical significance in this model.

**Figure 3:**
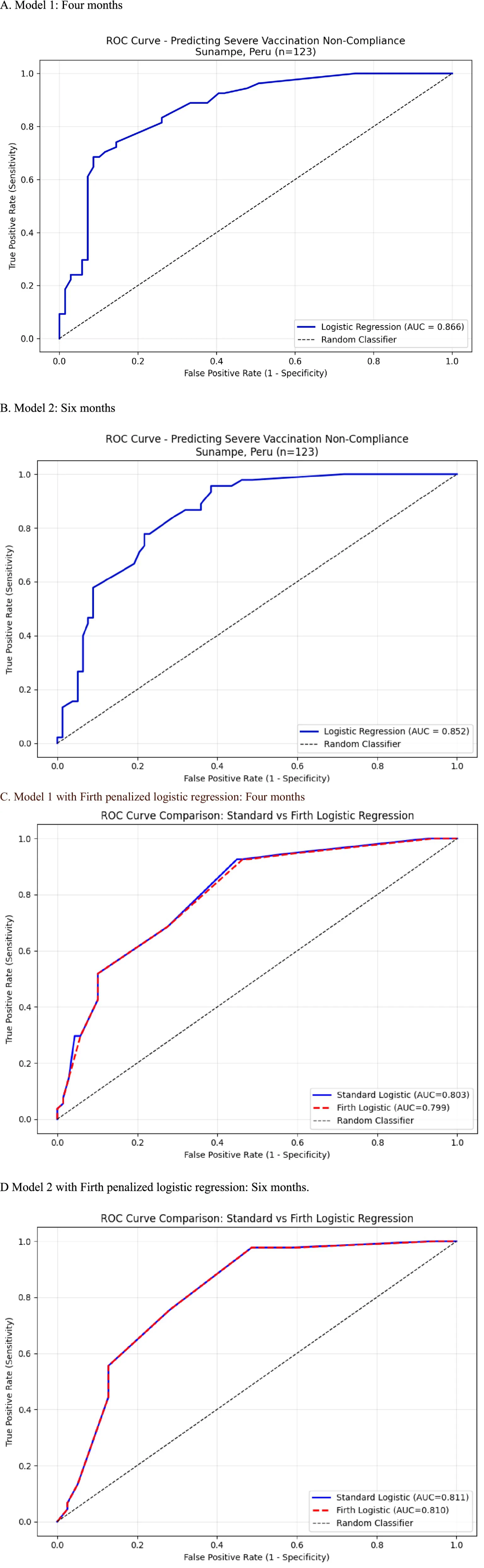
Receiver Operating Characteristic (ROC) curve for Model predicting severe vaccination non-compliance: **(A)** Model 1, stopped by 4 months, ≥2 missed doses, *n* = 54 severe, *n* = 69 mild. **(B)** Model 2, stopped by 6 months, ≥3 missed doses, *n* = 45 severe, *n* = 78 mild. **(C)** Model 1 with Firth penalized logistic regression: Four months. **(D)** Model 2 with Firth penalized logistic regression: Six months.

On the other hand, Table 3 (Model 1) presents the logistic regression model for severe non-compliance defined as vaccination stopped by 4 months (≥2 missed doses), alongside exploratory forward selection results (Supplementary Table S1). These analyses were exploratory and not used for primary inference (Figure 3).

Having only two variables, we explored Hub variables with additional analysis including forward selection as shown in Supplementary material. Supplementary Table S2 presents an exploratory inter-item association analysis examining which questionnaire items most frequently predicted other responses. Q22 (family objections to vaccination) was the most frequent predictor, associated with seven outcomes including early vaccination discontinuation (Q02, Q04, Q05) and access perceptions (Q09, Q10, Q12). Q17 (knowledge of vaccination age) predicted three outcomes (stopping at 6 months, living near a health center, and risk awareness), consistent with its strong performance in the main logistic regression model. Q18 (attendance at information sessions) predicted two outcomes (stopping at 4 months and waiting time inconvenience). Fear-related variables (Q20, Q21, Q23, Q25) did not appear as significant predictors in any model, supporting the main finding that fear does not drive non-compliance.

#### Sensitivity analysis: Firth’s penalized logistic regression

To address the low events-per-variable ratio (EPV = 4.5), we repeated the analysis using Firth’s penalized likelihood logistic regression with dimension reduction (knowledge score, fear score, access score). Results were consistent with the primary model: the knowledge score remained the strongest predictor (OR = 3.84), while fear and access scores did not reach statistical significance. The penalized model achieved an AUC of 0.810, nearly identical to the primary model (AUC = 0.811). These sensitivity analyses support the robustness of our findings despite the low EPV (Table 1 and calculi in Supplementary Table S1). Similar results were obtained for the 4-month threshold (Table 3).

### Disease trends

As shown in Table 4, the number of reported cases and deaths due to pertussis decreased before and during the pandemic. No comparable data were available for pneumonia and measles in this dataset.

**Table 4:** Cumulative number of cases and deaths due to different impairments on Peru.

Impairment	Age range	2016	2017	2018	2019	2020	2021	Reference
Whooping cough								
Cases	0–11 m	101	364	396	272	60	2	CNEPC
	1–4 years	23	97	97	132	17	0	CNEPC
Deaths	0–11 m	3	18	11	9	0	1	CNEPC
	1–4 years	0	0	0	3	0	0	CNEPC
Difteria								
Cases	0–11 m	0	0	0	0	1	N/A	CNEPC
	1–4 years	0	0	0	0	1	N/A	
Pneumonia								
	0–11 m							
	1–4 years							
Measles								
	0–11 m							
	1–4 years							

Extracted from: CDC-MINSA National Center for Epidemiology, Prevention and Disease Control–MINSA, until week 7 of 2021.

Although there was coverage during the pandemic, vaccine coverage was low as well as social distance measures.

It is noted that before the pandemic, there was a decrease in whooping cough with more cases.

After the pandemic, there was a decrease in the number of cases of whooping cough.

### Mortality analysis (deaths)

To contextualize the broader health landscape in Sunampe and Peru during the study period, we examined under-five mortality data from two sources. Table 5 compares under-five mortality data from IHME (2018) for the years 2000 and 2017 with national-level SINADEF records. In 2017, SINADEF reported 840 under-five deaths—substantially higher than the 311 deaths estimated by IHME. The highest number of deaths occurred in 2018, and death rates during the pandemic remained similarly high.

**Table 5:** Peruvian national data on deaths of children under 5 years of age, processed from SINADEF database.

Disease or impairment record	2000	2017	2018	2019	2020	2021	2022
HIV	8	23					
Meningitis	9	3					
Tuberculosis	10	2					
DPT	13	5					
Malnutrition	32	4					
Diarrhea	44	10					
Respiratory infection	163	47					
Neonatal disorder	234	100					
Others	298	117					
	811	311 ≠ 840	1203	1048	1157	1145	1191

These sources differ substantially in methodology: SINADEF reports raw counts from vital records, while IHME produces modeled estimates adjusted for underreporting and standardized to population. Direct comparison of raw figures between these databases is not methodologically valid, and no causal inference can be drawn from mortality data given the cross-sectional design of this study.

**Figure 4:**
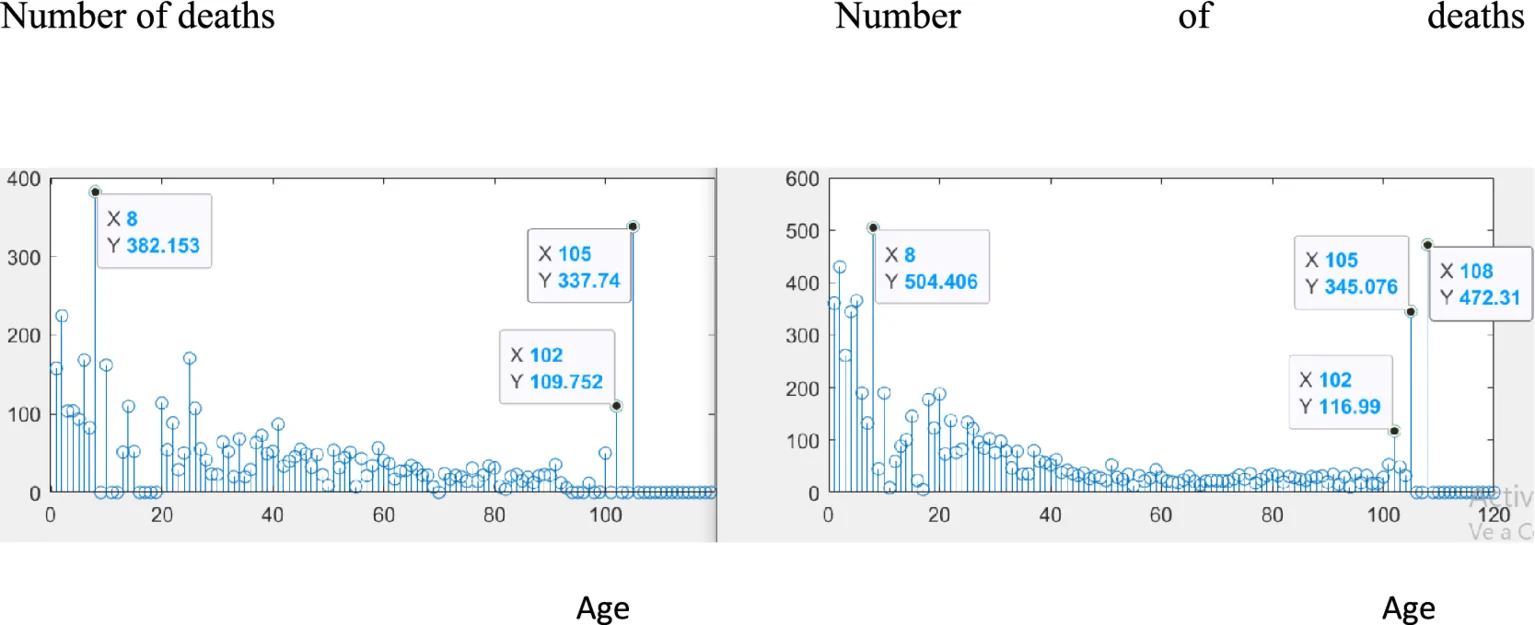
Pre-pandemic and pandemic pneumonia.

Figure 4 shows that children aged 2 years had the highest mortality rate, both before and during the pandemic. Elevated mortality was also observed in children aged 1, 4, and 5. This temporal association coincides with documented vaccination coverage declines that widened during the pandemic years, but causal inference is not possible due to ecological data and cross-sectional design.

### Age-specific mortality trends (Peru national data)

**Figure 5:**
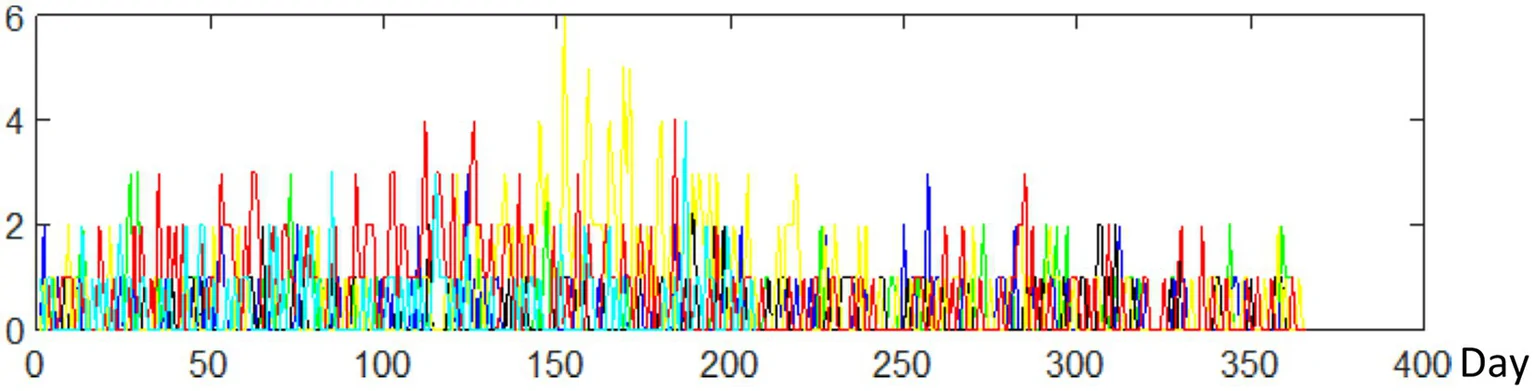
Number of deaths in children under five. Each color corresponds to a different year between 2017 and 2022 (in different colors).

In Sunampe, Figure 5 reveals a noticeable rise in mortality during 2020 (red) and 2021 (yellow), compared to the 2017–2019 baseline.

**Figure 6:**
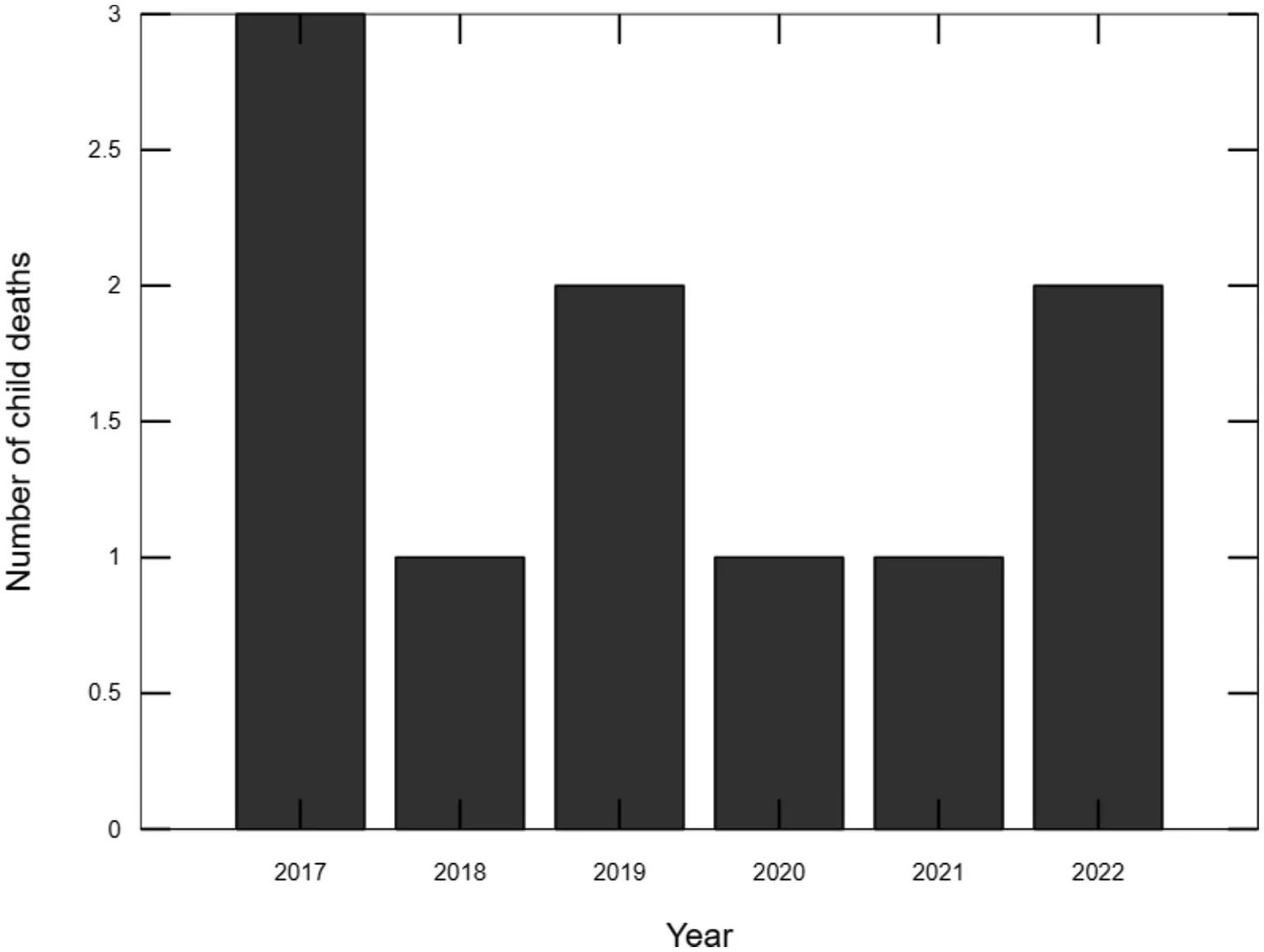
Number of deaths from vaccine-targeted diseases among children aged less than five in Sunampe. Each bar corresponds to a different year between 2017 and 2022.

SINADEF data filtered for Sunampe residents under 5 years of age show fewer than three deaths annually over the last 6 years (Figure 6). However, given the small population size, even a few deaths can significantly impact local public health assessments.

Data extracted from SINADEF between 2017 and 2022 revealed that 10 children under the age of five died in Sunampe, no one was recorded with anemia. Although 36 of 1072 death inhabitants with more than 5 years old were found with anemia.

An exploratory cause-of-death matching using keyword and ICD-10 code lists for vaccine-preventable diseases was performed on the same SINADEF records (Supplementary Table S3) having Pneumococcus as prevalent, therefore respiratory mechanics may be an issue. This analysis is purely descriptive and does not imply causation, as individual vaccination status was unknown and cause-of-death codes (e.g., sepsis) are non-specific. Among the 10 under-five deaths, none were directly coded as polio, measles, mumps, rubella, diphtheria, pertussis, or tetanus. However, extending Suarez’s analysis (24) to this period, four deaths had codes consistent with diseases potentially preventable by the pentavalent vaccine (DPT-HepB-Hib), and six had codes suggestive of pneumococcal disease (e.g., pneumonia, bronchopneumonia), that may have required intensive respiratory support (52). These counts are exploratory and do not imply that vaccination would have prevented those specific deaths.

### Risk: coverage, cases and deaths

The upper part of Table 6 shows the public data from the National Centre for Epidemiology, Prevention and Control of Diseases of Peru, comparing Ica with the whole of Peru. It is important to highlight that Ica, with 20.03%, has a lower susceptibility than the whole of Peru, with 27.05%.

**Table 6:** Difference in vaccination percentage of SP1 and SP2 SP1 and SP2 vaccination in Ica and Peru.

Ica	33298	6669	20.03%
Overall Peru	1107986	299762	27.05%

The data in the lower part of Table 6 show that the difference between SP1 and SP2 vaccination tends to be standardized with good or low coverage.

Limitations and improvements: Regarding the method, Hoest and colleagues (28) showed a method considering monthly and/or quarterly interviews, which would allow to see the vaccine compliance in monthly periods for 2 years. Considering that the selection of the children was based on the records of the health center in Hoest’s and the present study, an improvement of the methods and results can be done in the future.

The accuracy, although it depends on the ability and willingness of the parents to give an accurate answer, is present in the research methods based on interviews. In order to improve the significance of the work, an information crossing was carried out. Moreover, convenience sample based on available records from the single health center; no *a priori* power calculation was performed.

The mortality data presented in this section serve only to describe the health context of the study population. The following limitations preclude any causal inference:

No causal model linking vaccination status to mortality was tested.

Cross-sectional design cannot establish temporal sequence or causation.

Ecological fallacy risk: Aggregated mortality data do not reflect individual-level outcomes.

Small numbers in Sunampe (≤3 deaths/year) preclude statistical modeling.

IHME vs. SINADEF methodological differences mean raw counts are not directly comparable.

No death had anemia listed as a primary or contributing cause. This finding is consistent with the nature of death certificate data, which typically records the immediate medical cause of death (e.g., sepsis, respiratory failure) and not underlying conditions such as anemia.

Counts of deaths potentially related to missing vaccination (heuristic, not causal).

Therefore, these mortality data are presented as context, not as evidence of vaccine impact.

## Discussion

This study highlights several critical factors affecting vaccination compliance among parents in Sunampe, Peru. Among 123 children with incomplete vaccination histories, 45 (36.6%) had discontinued vaccination by 6 months of age. Logistic regression revealed that parents who did not know the appropriate vaccination age were nearly 11 times more likely to have severely non-compliant children (OR = 10.96, 95% CI: 3.40–35.36, *p* = 0.0001). Additionally, parents who had never attended a vaccine information session were 3.3 times more likely to discontinue vaccination early (OR = 3.32, 95% CI: 1.16–9.57, *p* = 0.026). This finding is consistent with other studies, such as that by Yalcin et al. in Turkey (29), which emphasizes the effectiveness of interpersonal strategies in countering vaccine hesitancy. Strengthening communication can reduce the risk of outbreaks of vaccine-preventable diseases, especially among children under 5 years of age—a demographic particularly vulnerable due to delayed or missed immunizations.

### Vaccination schedule

Data from this study (Table 2A) show that a significant majority (93.5%) of children with incomplete vaccination schedules are covered by public health insurance, indicating that economic cost is not the primary barrier. This contrasts with findings by Alker et al. (30), who noted a decline in insured Latino children under five in the U.S. to below 92%. However, healthcare access and infrastructure quality differ considerably between Peru and the U.S. In Peru, despite near-universal public insurance, systemic issues like healthcare delivery delays and limited facilities increase the burden of illness and hospitalization (31).

On the other hand, in Sunampe-Peru, this study found that 93.5% of the mothers interviewed had public insurance (SIS or ESSALUD) and 89.4% had a partner (married or cohabiting). These figures in Peru are similar to those of Latinos in the US, but they are low given the level of primary health care and the fragility of the progress made in the face of the pandemic.

Unlike Tambo Mora—which has five health institutions for a population of around 10,000—Sunampe, with a single clinic for a population of 30,000 (including 2,500 children under five), faces a significant disadvantage. This lack of infrastructure directly contributes to vaccination delays and incomplete schedules.

### Timely administration of vaccines

The results in Table 2B indicate serious gaps in timely vaccine administration. Only 57 of the 99 children received their first measles-mumps-rubella (SPR 1) vaccine as scheduled. This aligns with the findings of Hernández et al. (2), who reported high dropout rates between doses and an overall national coverage rate of only 48.9%. Similarly, Hoest et al. (28) found significant age-related delays in measles vaccination.

In Peru, Carhuavilca (32) found that only 56.9% of children under 1 year followed the vaccination schedule, with irregular adherence among 46.1%. These results underscore the need for targeted strategies to close the immunization gap, ensure timely protection, and prevent vaccine-preventable diseases. Accurate documentation—via vaccination cards, health center records, or digital systems—is crucial for verifying immunization status and planning effective interventions.

### Access to vaccination appointments

Structural issues, such as long wait times and a lack of sufficient health facilities, were reported by the majority of families. These service-related shortcomings often led parents to seek vaccination services in neighboring districts, further complicating adherence to local immunization schedules. Survey responses in Table 2C reveal that 91.06% of mothers consider the waiting time for appointments excessive, while 82.9% report difficulties securing appointments, and 95.1% believe there are too few facilities. This matches the findings of Ekhaguere et al. (33), who observed waiting times ranging from 57 to 235 min depending on the health center. Complex administrative processes further exacerbate these delays.

Given that Sunampe has only one vaccination center for 2,500 children under five, expanding infrastructure is essential. Streamlining vaccine delivery and administrative protocols is critical to improving adherence and avoiding missed vaccination opportunities. From a clinical decision making perspective, this means ensuring that every contact with the health system is used to verify vaccination status and provide tailored education, especially in settings with limited infrastructure.

### Parental knowledge of vaccines

The 6-month threshold identified two significant predictors (Q17, Q18), while the 4-month threshold only identified Q17. Q18 became significant when severe non-compliance was defined more stringently, suggesting that lack of information sessions is particularly important for preventing early discontinuation, moreover in Supplementary material explored Q17 and Q18 as hub variables. Although most mothers acknowledged the importance of vaccines, 87.8% were unaware of the number of vaccines required, and 99.2% did not know the diseases they prevent. These findings mirror those from Bangura et al. (34), who identified limited knowledge as a key barrier. Stephanie et al. (35) also found that even when awareness of specific diseases was low, parents still valued vaccines.

Of note, Q18 only became significant when severe non-compliance was defined as stopping by 6 months (our primary model). In the 4-month threshold model (see Table 3), Q18 had borderline significance (*p* = 0.053). This suggests that information sessions are particularly important for preventing discontinuation during the critical 4–6 month window, when infants receive pentavalent and polio vaccines.

The consistency of the knowledge score as the sole significant predictor across both thresholds (4-month and 6-month) and across both estimation methods (standard and Firth penalized logistic regression) strengthens our conclusion that knowledge gaps, not fear of reactions or access barriers, are the primary modifiable drivers of severe vaccination non-compliance in Sunampe.

Our finding that knowledge gaps drive severe non-compliance is consistent with large-scale studies from India, where delayed BCG, DPT-1, and measles were associated with lower maternal education and poorer household wealth (21). Across 45 low- and middle-income countries, substantial proportions of children receive vaccines weeks or months after the recommended age (19), and high reported coverage often masks these delays (20). Importantly, the global EPI has averted an estimated 154 million deaths since 1974, with 40% of the decline in infant mortality attributed to vaccination, and 52% in the African region (22). Our results reinforce that improving timeliness, not just coverage, requires addressing parental knowledge and access to information sessions.

In Peru, Samame (36) noted similar trends, citing ignorance as a primary cause of non-compliance. Cultural factors further complicate vaccine uptake, as highlighted by Carhuavilca (32). These results emphasize the vital role of healthcare professionals in educating caregivers and aligning public health efforts with national immunization goals.

Zhang et al. (37) found that in China, COVID-19 vaccine hesitancy among parents of children aged 3–11 was linked to low health knowledge, particularly among mothers and parents of allergic children. This parallels our findings and suggests that recent global events may have altered parental attitudes toward vaccines. One can consider whether COVID-19 vaccine policies influenced perceptions of routine immunization in Sunampe. Our study, coupled with this finding, prompts further consideration regarding changes in parental attitudes due to the recent pandemic.

National-level trends in disease incidence are shown in Table 2C, where pertussis cases declined from 272 (pre-pandemic) to 60 (2021). This decline may reflect reduced exposure due to COVID-19 restrictions, consistent with observations in Spain (38). Similarly, in England, Public Health England data showed a reduction in pertussis notifications during the first national lockdown compared to pre-pandemic averages (39). In the United States, CDC surveillance reported a decline in pertussis cases in 2020–2019 (40).

#### Vaccine reactions and compliance

Logistic regression analysis showed low AUC values, indicating poor predictive accuracy for vaccine-related responses. However, Q13 (sufficiency of vaccination centers) was a statistically significant predictor of Q25 (whether a child had an allergic vaccine reaction), although this should be interpreted cautiously due to low model performance.

Table 2E reveals that 99.2% of mothers expressed fear of vaccine reactions, a key barrier to compliance despite recognizing the health benefits of vaccination. Samame (36) noted similar fears, including documented adverse events such as the 2010 influenza vaccine reactions and a 2015 whooping cough-related death.

#### Vaccination coverage and mortality

Table 2F shows that 123 children remained incompletely vaccinated over 5 years, with the worst rate in 2017 (23.94%) and a partial rebound to 43.89% in 2021. These results are consistent with Hernández et al. (2), who found a 48.9% complete schedule coverage in Mexico, with indications of overestimation in official reports.

In Sunampe, the group most at risk are one-year-olds. In 2020, their vaccination rate was only 43.17%, close to the 47.59% rate in Chincha. These low rates indicate persistent systemic challenges.

Child mortality analysis over 6 years using SINADEF data showed deaths from causes that may be preventable through public health interventions, then those few vaccine-preventable deaths annually, but these could be underreported. While the majority of deaths were not directly linked to vaccination gaps, preventable deaths may still occur. Our findings align with Xu et al. (18) and Wolff et al. (41), who identified sociodemographic disparities in child mortality. In the future, one may wish to explore how socioeconomic and ethnic disparities influence both vaccination rates and health outcomes in Sunampe.

The majority of cases are caused by factors other than vaccination. The SINADEF database was used to study data in children over a period of 6 years. Although most cases were not linked to vaccine preventable diseases, there were still a few cases reported annually in Sunampe, a small community in Peru. It is therefore feasible to explore other possible cofactors as identified in the SINADEF report. Our findings describe mortality trends; no causal link to vaccination status was established.

The finding that six of the ten deaths were from causes suggestive of severe pneumococcal disease (pneumonia/sepsis) is clinically significant. In young children, such infections can rapidly progress to respiratory failure, often necessitating mechanical ventilation. The unique respiratory mechanics of infants and young children—including smaller airways and lower functional residual capacity—make them particularly vulnerable to hypoxia and way to avert this cascade of life-threatening events.

#### Limitations

Because the study enrolled only children with incomplete vaccination histories, the analysis compares degrees of non-compliance (severe vs. mild) and does not evaluate determinants of complete vaccination versus any vaccination in the general pediatric population. Consequently, our findings apply to varying severity of non-adherence among already under-vaccinated children and cannot be extrapolated to fully compliant children. Nevertheless, the associations we identified (knowledge of age, information session attendance) are biologically plausible and consistent with predictors of delayed vaccination reported in national surveys from other low- and middle-income countries (19, 21).

Additionally, adverse reaction fears, although mostly unfounded, still limit vaccine compliance. Wilkins et al. (42) reported no significant adverse events with new influenza vaccines, supporting this conclusion. One can consider stating whether Sunampe follows a good coverage in terms of population according to fellows and workers in the health center. Therefore, systematic protocols in vaccine administration considering social and economics issues around Sunampe.

Wolff et al. (41) reported that younger ages correlated with increased likelihood of hospital death, while non-white or low-income children, as well as those with non-oncology diagnoses, were less likely to pass away at home compared to their counterparts who were white, had high incomes, or had oncology diagnoses. Income and oncology diagnoses were not explored in the present research.

Additionally, vaccine hesitancy was identified as a limitation due to concerns about adverse effects. An example is provided concerning the administration of various influenza vaccines at a health center with a mostly randomized approach to immunoprevention. Wilkins et al. (47) determined that there were no significant incidents of serious adverse events associated with the use of new influenza vaccines when compared to control vaccines. However, a project proposal may assist in mitigating cognitive impairments (41, 43). It is also possible that a generic DPT vaccine will become available in the near future.

Despite the high national prevalence of childhood anemia [~43%, (21, 51)] and its known link to increased infection risk and mortality, none of the ten under-five deaths in Sunampe were directly attributed to anemia in the SINADEF database. This paradox likely arises because death certificates prioritize immediate fatal events; however, it is crucial to note that future work may seek if anemia is a well-established cofactor that can increase the risk and severity of fatal outcomes.

## Conclusion

This study highlights persistent public health vulnerabilities in Sunampe, Peru, where, despite isolated improvements, under-five mortality remains a concern and national trends show little change. Although most mothers expressed a belief in the value of vaccination, widespread gaps in knowledge and awareness persist, contributing to fear, non-compliance, and incomplete immunization schedules.

Logistic regression identified two significant predictors of severe non-compliance. Parents who did not know the appropriate vaccination age were nearly 11 times more likely to have severely non-compliant children (OR = 10.96, 95% CI: 3.40–35.36, *p* = 0.0001). Parents who had never attended a vaccine information session were 3.3 times more likely to discontinue vaccination early (OR = 3.32, 95% CI: 1.16–9.57, *p* = 0.026). The model showed good discriminative ability (AUC = 0.852, accuracy = 76.4%).

Contrary to initial assumptions, the majority of parents reported no serious adverse vaccine reactions, and many who delayed or skipped vaccination later expressed regret. These findings suggest that non-compliance is more strongly associated with knowledge interventions (information sessions, vaccination age education and inadequate information, rather than with the direct experience of vaccine-related complications).

The findings of this study underscore the importance of addressing both informational and logistical barriers to immunization. Targeted public health messaging, combined with expanded healthcare infrastructure, may help to reduce fears and improve vaccine uptake. Ultimately, these results provide actionable evidence to guide clinical decision making and health policy in similar resource‑limited settings.

Despite the high national prevalence of childhood anemia (~43%), none of the ten under-five deaths in Sunampe were directly attributed to anemia. This likely reflects death certificate coding practices; however, anemia remains an important co-morbidity that may increase vulnerability to infections and should be addressed in future research.

### Future directions

To build upon this study:

A validated questionnaire was administered to mothers of 123 children under five with incomplete vaccination records.

Epidemiological data on illness and mortality in Sunampe were analyzed alongside national health records.

While child mortality in Sunampe remained low (no more than three deaths annually), national data showed no significant decline, raising concerns about broader immunization gaps.

Future research should evaluate the effectiveness of knowledge-transfer interventions (e.g., community health worker home visits, mobile messaging) in large randomized trials. Furthermore, for improving clinical decision making, investigating the interplay between vaccination and nutritional co-factors like anemia is vital, given that >40% of Peruvian children remain at risk, and their mothers as well (43). Such longitudinal, large-scale studies are essential to address epidemiological vulnerability (46) while exploring the evolving health interventions (48–50) and country productivity (44, 45) also in pediatric populations.

## Data Availability

The datasets presented in this study can be found in online repositories. The names of the repository/repositories and accession number(s) can be found at: https://github.com/cmugruza/Vaccine_Deaths_Sunampe.

## Footnotes

1
https://cloud.minsa.gob.pe/s/NctBnHXDnocgWAg?path=/&scrollto=SINADEF_DATOS_ABIERTOS.xlsx

2
https://github.com/cmugruza/Vaccine_Deaths_Sunampe
