# Supplementary material for: Understanding vaccination gaps in Peru: a convergent cross-sectional analysis of coverage, parental perceptions, and child mortality data in Sunampe
Source: Front Public Health. 2026 Aug 26;14:1779442. doi: 10.3389/fpubh.2026.1779442 (PMC13553873; doi:10.3389/fpubh.2026.1779442)
Supplement: Supplementary file 1 [file Supplementary_file_1.docx]

**Understanding Vaccination Gaps in Peru: A Convergent Cross-Sectional Analysis of Coverage, Parental Perceptions, and Child Mortality Data in Sunampe**

SUPPLEMENTARY MATERIAL

# Introduction

To identify factors associated with vaccine non-compliance, forward selection logistic regression was applied to 25 survey questions (Q1–Q25), each treated as a dependent variable, using a p-value threshold of 0.05.

Logistic regression models had fair to good discriminative ability (0.70-0.91low AUC values). However, forward selection revealed significant associations among questionnaire items, notably family objections to vaccination (Q22), early vaccination discontinuation (Q02–Q05), ease of access (Q09), and parental knowledge of immunization schedules (Q14, Q17).

Hub variables were identified: Q22 (family objections to vaccination), Q18 (information session attendance). Q17 (knowledge of vaccination age) and Q14 (knowledge of vaccine quantity).

# Methods

A series of 25 logistic regression models were constructed to analyze the factors associated with responses to each question (Q01-Q25). The analysis aimed to identify significant predictors for each response in Supplementary Table 1.

Across the 25 logistic regression models, only a small number exhibited statistically significant predictors (p < 0.05). Notably, forward selection approach revealed inter-item associations within the questionnaire, highlighting underlying constructs and potential explanatory patterns of parental attitudes and behaviors toward vaccination with Q22 (Does anyone in your family object to your child being vaccinated?), Q02-Q05 (Did your child get vaccinated only up to a few months?) , Q09 (Did you find it easy to get vaccinated at the Health centre?), Q14 (Do you know how many immunizations through vaccines your child should have?) and Q17 (Do you know at what age your child should be vaccinated?). The forward selection analysis revealed significant inter-item associations across multiple questions. For example, Q02 was significantly predicted by Q03 (p = 0.00, AUC = 0.83), Q05 (p = 0.00, AUC = 0.72), Q09 (p = 0.01, AUC = 0.82), and Q22 (p = 0.02, AUC = 0.84). Similar predictive relationships were observed for other items, such as Q12 being predicted by Q03, Q11, Q19, and Q22, with consistently high AUC values above 0.85. These patterns suggest overlapping constructs among questions, reflecting the multifaceted reasons for non-compliance, including perceptions of vaccine safety, access barriers, and informational gaps. The models exhibited good discriminative ability, with AUC values ranging from 0.70 to 0.91 and classification accuracy often exceeding 0.80 (see Supplementary Figure 1).

Supplementary Table S1. Exploratory forward selection logistic regression results showing inter-item associations among questionnaire items (n=123).Results of Logistic Regression

| **Target** | **Predictor** | **P-Value** | **AIC** | **AUC** | **Accuracy** |
| --- | --- | --- | --- | --- | --- |
| Q02 | Q05 | 0.00 | 150.67 | 0.72 | 0.72 |
| Q02 | Q03 | 0.00 | 134.00 | 0.83 | 0.84 |
| Q02 | Q09 | 0.01 | 127.90 | 0.82 | 0.86 |
| Q02 | Q22 | 0.02 | 124.29 | 0.84 | 0.85 |
| Q03 | Q02 | 0.00 | 142.01 | 0.72 | 0.71 |
| Q03 | Q12 | 0.00 | 131.93 | 0.78 | 0.72 |
| Q03 | Q18 | 0.01 | 125.81 | 0.81 | 0.76 |
| Q04 | Q17 | 0.00 | 146.43 | 0.73 | 0.72 |
| Q04 | Q05 | 0.00 | 135.95 | 0.81 | 0.79 |
| Q04 | Q14 | 0.03 | 132.08 | 0.83 | 0.80 |
| Q04 | Q22 | 0.02 | 128.35 | 0.85 | 0.80 |
| Q04 | Q09 | 0.02 | 124.40 | 0.85 | 0.80 |
| Q05 | Q02 | 0.00 | 150.02 | 0.72 | 0.72 |
| Q05 | Q04 | 0.00 | 142.28 | 0.78 | 0.82 |
| Q05 | Q22 | 0.03 | 139.06 | 0.79 | 0.76 |
| Q07 | Q17 | 0.00 | 161.33 | 0.63 | 0.62 |
| Q07 | Q14 | 0.04 | 158.67 | 0.68 | 0.65 |
| Q07 | Q05 | 0.04 | 156.32 | 0.72 | 0.69 |
| Q07 | Q03 | 0.04 | 153.94 | 0.72 | 0.73 |
| Q09 | Q02 | 0.00 | 102.63 | 0.71 | 0.83 |
| Q09 | Q22 | 0.02 | 98.53 | 0.78 | 0.83 |
| Q09 | Q04 | 0.02 | 94.76 | 0.82 | 0.84 |
| Q10 | Q22 | 0.00 | 155.97 | 0.65 | 0.63 |
| Q10 | Q08 | 0.01 | 149.42 | 0.70 | 0.67 |
| Q12 | Q03 | 0.00 | 72.66 | 0.77 | 0.89 |
| Q12 | Q22 | 0.02 | 68.36 | 0.84 | 0.89 |
| Q12 | Q11 | 0.02 | 65.56 | 0.88 | 0.89 |
| Q12 | Q19 | 0.04 | 60.54 | 0.91 | 0.89 |
| Q14 | Q04 | 0.00 | 83.14 | 0.73 | 0.88 |
| Q14 | Q07 | 0.01 | 77.39 | 0.82 | 0.88 |
| Q14 | Q24 | 0.01 | 73.12 | 0.84 | 0.89 |
| Q17 | Q04 | 0.00 | 145.26 | 0.73 | 0.72 |
| Q17 | Q19 | 0.00 | 130.71 | 0.81 | 0.77 |
| Q17 | Q07 | 0.00 | 123.57 | 0.85 | 0.77 |
| Q18 | Q03 | 0.00 | 143.28 | 0.64 | 0.71 |
| Q18 | Q12 | 0.03 | 140.44 | 0.67 | 0.72 |
| Q19 | Q17 | 0.00 | 140.60 | 0.72 | 0.69 |
| Q19 | Q02 | 0.01 | 136.53 | 0.77 | 0.72 |
| Q22 | Q10 | 0.00 | 162.50 | 0.64 | 0.63 |
| Q22 | Q18 | 0.02 | 158.43 | 0.70 | 0.68 |
| Q22 | Q09 | 0.03 | 155.39 | 0.72 | 0.69 |
| Q22 | Q04 | 0.02 | 151.20 | 0.76 | 0.72 |
| Q24 | Q14 | 0.02 | 53.23 | 0.66 | 0.94 |

Supplementary Figure 1. AUC by forward selected predictors.

Supplementary table 1 shows inter-item associations from forward selection. However, these results actually validate why Q17 and Q18 emerged as significant in the corrected logistic regression (Figure 3 and Table 3). Therefore Q17 and Q18 are "Hub" Variables, as Supplementary Table 2 presented, often each predictor appears.

Supplementary Table S2. Frequency of significant associations (p < 0.05) among questionnaire items from forward selection logistic regression.

| **Predictor** | **Appears As Predictor For** | **Interpretation** |
| --- | --- | --- |
| Q17 | Q04, Q07, Q19 (3 times) | Central knowledge hub - predicts early stopping (Q04), access perception (Q07), and risk awareness (Q19) |
| Q18 | Q03, Q12 (2 times) | Information-seeking hub - predicts stopping at 4 months (Q03) and waiting time perception (Q12) |
| Q22 | Q02, Q04, Q05, Q09, Q10, Q12, Q18 (7 times) | Family objections - but dropped from your final model due to collinearity with Q17/Q18 |
| Q14 | Q04, Q07 (2 times) | Knowledge of vaccine quantity - significant in bivariate but not in multivariate (suppressed by Q17) |

Therefore, Q17 (Knowledge of vaccination age) is the strongest predictor in tables: Q17 predicts Q04 (stopping at 6 months), Q07 (living near the health centre), and Q19 (risk awareness). This means parents who don't know vaccination ages are more likely to stop early, live far from clinics, and are unaware of disease risks. Also Q17 gets some of the explanatory power of Q14 and Q16 in multivariate models

Moreover, Q18 (Attended information session) is the second strongest: Q18 predicts Q03 (stopping at 4 months) and Q12 (waiting time inconvenience). This means parents who never attended sessions are more likely to stop early and perceive access barriers. Also, Q18 captures the effect of health system outreach that Q09, Q11, Q12 cannot measure alone.

In addition Q22 (Family objections) is correlated with Q17/Q18: Q22 predicts 7 different outcomes but did not reach significance in your multivariate model This suggests family objections operate through knowledge gaps (Q17) and lack of information (Q18)In other words: families who object to vaccines also tend to have lower knowledge and never attend sessions

Finally, fear variables (Q20, Q21, Q23, Q25) are weak predictors: These do not appear in your table as significant predictors. This confirms that fear is not a direct driver of non-compliance in the sample. Parents report fear (99.2%), but fear does not predict behavior in multivariate models

Our inter-item association analysis (Table 3, original version) revealed that knowledge-related variables (Q17, Q18) and family objections (Q22) were the most frequent predictors of early vaccination discontinuation. However, in multivariate analysis, only Q17 (knowledge of vaccination age) and Q18 (attendance at information sessions) remained significant. Family objections (Q22) did not reach significance, suggesting that its effect is mediated through knowledge gaps—families who object to vaccines also tend to lack accurate information about vaccination schedules.

Notably, fear-related variables (Q20, Q21, Q23, Q25) did not appear as significant predictors in any model. This apparent paradox, 99.2% of mothers reported fear of reactions, yet fear did not predict severe non-compliance—suggests that reported fear may reflect social desirability bias or general anxiety rather than a true barrier to vaccination. Alternatively, fear may be a necessary but not sufficient condition for non-compliance, requiring the additional presence of knowledge gaps or access barriers to manifest as behavior.

On the other hamd, to explore potential missed preventive opportunities, following Suarez et al analysis [55], we applied a heuristic keyword and ICD‑10 code matching algorithm to the cause‑of‑death fields of the 10 under‑five deaths recorded in SINADEF. The algorithm searched for terms related to diseases targeted by the five priority vaccines (APO, pentavalent, pneumococcus, SPR, DPT booster). This analysis is exploratory and does not establish causality. Individual vaccination status was not available, and causes such as sepsis or pneumonia can be due to non‑vaccine‑preventable pathogens.

Supplementary Table S3. Death causes matched by missing priority vaccine

Cohort: 4-59 Months Old in the District of Sunampe at the 2017-2021.

|  | **Death causes** | **Possible missing priority vaccine** |
| --- | --- | --- |
|  | 0 | APO (Oral Antipolio) |
|  | 4 | Pentavalent (DPT-HepB-Hib) |
|  | 6 | Pneumococcus |
|  | 0 | SPR (Measles, Mumps, Rubella) |
|  | 0 | DPT Booster (Diphtheria, Pertussis, Tetanus) |

These counts are presented for hypothesis‑generation only. They do not imply that vaccination would have prevented these specific deaths, nor do they estimate vaccine effectiveness.

An additional limitation is that the SINADEF database does not contain detailed clinical information, such as the need for mechanical ventilation or the specifics of respiratory support. Therefore, we cannot confirm that the six deaths potentially linked to pneumococcal disease actually required intensive respiratory support. This represents a gap in our ability to fully characterize the clinical trajectory of children's deaths.

# Conclusions

The exploratory inter‑item association analysis (Supplementary Tables S1–S2) revealed statistically significant relationships among several questionnaire items, indicating overlapping conceptual domains in parental beliefs and behaviors related to vaccination non‑compliance in Sunampe, Peru.

Hub variable identification was suggested from the supplementary analysis, as Q22 (family objections to vaccination) was the most frequent predictor, associated with seven outcomes including early vaccination discontinuation (Q02, Q04, Q05), access perceptions (Q09, Q10, Q12), and information session attendance (Q18). Q17 (knowledge of vaccination age) predicted three outcomes (stopping at 6 months Q04], living near a health centre Q07, and risk awareness Q19), consistent with its strong performance in the main logistic regression model (Table 3). Q18 (attendance at information sessions) predicted two outcomes (stopping at 4 months Q03 and waiting time inconvenience Q12).

Failure of fear variables were suggested from the supplementary analysis, as fear‑related items (Q20, Q21, Q23, Q25) did not appear as significant predictors in any exploratory model. This finding corroborates the main analysis: although 99.2% of mothers reported fear of vaccine reactions, fear does not predict early vaccination discontinuation.

The finding that six of the ten deaths were from causes suggestive of severe pneumococcal disease (pneumonia/sepsis) is clinically significant. In young children, such infections can rapidly progress to respiratory failure, often necessitating mechanical ventilation. The unique respiratory mechanics of infants and young children, including smaller airways and lower functional residual capacity, make them particularly vulnerable to hypoxia and hypercapnia. This underscores the importance of pneumococcal vaccination as a primary prevention strategy, as preventing the initial infection is the most effective way to avert this cascade of life-threatening events.
